# Growth of MoS_2_ Nanosheets on Brush-Shaped PI–ZnO Hybrid Nanofibers and Study of the Photocatalytic Performance

**DOI:** 10.3390/nano15010044

**Published:** 2024-12-30

**Authors:** Zhenjun Chang, Zhengzheng Liao, Jie Han, Qiang Liu, Xiaoling Sun

**Affiliations:** 1 College of Materials Science and Engineering, Jiangsu University of Science and Technology, Zhenjiang 212003, China; 2 Polytex Engineering Group, Yangzhou 225000, China

**Keywords:** nanofibers, hybrid, electrospinning, polyimide, ZnO, MoS_2_

## Abstract

The design and preparation of advanced hybrid nanofibers with controllable microstructures will be interesting because of their potential high-efficiency applications in the environmental and energy domains. In this paper, a simple and efficient strategy was developed for preparing hybrid nanofibers of zinc oxide–molybdenum disulfide (ZnO–MoS_2_) grown on polyimide (PI) nanofibers by combining electrospinning, a high-pressure hydrothermal process, and in situ growth. Unlike simple composite nanoparticles, the structure is shown in PI–ZnO to be like the skeleton of a tree for the growth of MoS_2_ “leaves” as macro-materials with controlled microstructures. The surface morphology, structure, composition, and photocatalytic properties of these structures were characterized using scanning electron microscopy, X-ray diffraction, X-ray photoelectron spectroscopy, and UV–vis spectroscopy. The ultra high-volume fraction of MoS_2_ can be grown on the brush-shaped PI–ZnO. Decorating ZnO with nanosheets of MoS_2_ (a transition metal dichalcogenide with a relatively narrow band gap) is a promising way to increase the photocatalytic activity of ZnO. The hybrid nanofibers exhibited high photocatalytic properties, which decomposed about 92% of the methylene blue in 90 min under visible light irradiation. The combination of MoS_2_ and ZnO with more abundant surface-active sites significantly increases the spectral absorption range, promotes the separation and migration of carriers, and improves the photocatalytic characteristics.

## 1. Introduction

Due to the acceleration of the world’s industrialization, environmental issues have arisen [1]. Organic dyes have been widely used in water-based plastics, papermaking, textiles, and other chemical industries. They may cause skin diseases, respiratory infections, eye irritation, and other chronic effects on organisms. Therefore, the degradation of the toxic chemicals output by industry is necessary for environmental pollution control. Photocatalysis is a promising way to remove organic components from wastewater. Semiconductor metal oxides (e.g., TiO_2_, ZnO, CeO_2_, WO_3_), precious metals (e.g., Ag, Au, Pt, etc.), and metal chalcogenides (e.g., ZnS, MoS_2_, WS_2_) have attracted great attention due to their photocatalytic performance [2,3].

ZnO has attracted great attention due to its high surface reactivity, environmental friendliness, high photosensitivity, low price, and chemical stability. When ZnO particles are irradiated with ultraviolet light (<400 nm), they generate strong oxidants—hydroxyl radicals, which can degrade organic pollutants in wastewater [4]. However, due to its photocorrosive effect, the stability of the photocatalyst is reduced, and it is only deteriorated when exposed to ultraviolet rays, so there are problems, such as a wide band gap energy [5]. In addition, due to high recombination rate of the electron–hole (e–h) pairs, the use of pure ZnO as a photocatalyst is fundamentally limited. Therefore, it is very important to improve the separation efficiency of the e–h pairs to improve the photocatalytic activity of ZnO. Several studies have been conducted to improve the photocatalytic activity of ZnO and suppress the e–h pair recombination rate by linking them with other suitable materials such as low-band semiconductors, precious metals, and carbon materials [6,7,8]. In addition, ZnO particles are biosafety materials, which have attracted attention not only for their photocatalytic activity but also for their antibacterial activity.

Graphene has caused a great sensation in chemistry, physics, materials science, and related fields. Recently, other layered materials such as molybdenum disulfide (MoS_2_) have been investigated for a variety of applications due to their unique physical properties [9]. In addition, two-dimensional (2D) MoS_2_, which has a layered structure, has been widely studied as a promising material due to its excellent electrical, chemical, and optical properties [10,11]. For example, Zhang et al. synthesized layered MoS_2_ shells loaded onto carbon spheres using the one-step hydrothermal method, showing a high specific capacity, enhanced cyclic stability, and a good multiplier performance [12]. Some groups prepared the ZnO/PVA: MoS_2_ stacked structure and MoS_2_@ZnO heterostructures [13,14]. We prepared a hierarchical structure of TiO_2_ nanorods with MoS_2_ nanosheets [15,16].

MoS_2_ is one of the metal dichalcogenide groups belonging to quasi-two-dimensional materials. The existence of a weak van der Waals gap between these atomic layers is responsible for the photoetching, chemical inertance, lubrication, catalysis, and optical properties of MoS_2_. Nanosized MoS_2_ has more active sites than bulk MoS_2_ due to its direct band gap. Bulk MoS_2_ has an indirect band gap. The enhanced photocatalytic activity of nanoscale MoS_2_ is attributed to its small direct band gap (1.7 eV), wide absorption range of 400~700 nm, and sufficient BET surface area [17]. Nevertheless, it becomes relatively unstable in water, which guides the lattice to dissolve via the oxidation of sulfur into sulfate ions. However, it does not affect the photocatalytic activity because this process is very slow. In addition, the charge separation insufficiency and low charge mobility are other disadvantages of MoS_2_. To solve this problem, it is always used in heterogeneous structures combined with other semiconductors in order to reduce the recombination of the e–h pairs, with the possibility of enhancing the charge separation capabilities. As a result, many heterogeneous structures such as MoS_2_/graphene [18] and MoS_2_/WS_2_ [19] have been manufactured for different applications, such as the H_2_ release and photovoltaic performance in lithium-ion batteries. For the purpose of better control of MoS_2_ loading and effective dispersion, it is still very desirable to find a skeleton to immobilize them and further make them easy for facile catalyst recovery and recycling.

However, there are seldom studies on the preparation and application of MoS_2_/ZnO immobilized nanofibers [20,21]. The brush nanorod–polymer nanofibers (a large surface area) have a membrane structure, which will act as an excellent skeleton for immobilization of the nanoparticles or nanosheets with high loading for functional application. In this paper, we used a two-step hydrothermal method to synthesize a ZnO–MoS_2_ composite at high temperature. In this study, electrospun polyimide (PI) nanofibers were used as the skeleton. The brush-like PI–ZnO nanofibers attached to the MoS_2_ nanosheets were successfully synthesized using the two-step hydrothermal method. The MoS_2_ nanosheets were uniformly dispersed and attached to the brush-like PI–ZnO surface. Here, the high specific area of the brush-shaped nanofibers provides sufficient surface for the growth of MoS_2_, which makes the charge separation more effective and reduces the possibility of the recombination of the e–h pairs, resulting in enhanced photocatalytic activity.

## 2. Materials and Methods

### 2.1. Materials

The pyromellitic dianhydride (PMDA) and 4,4′-oxydianiline (4,4′-ODA) were of chemical reagent grade and purchased from Sinopharm Chemical Reagent Co., Ltd. (Shanghai, China); they were further purified using sublimation before use. The zinc acetate dehydrate (Zn(Ac)_2_·2H_2_O), zinc nitrate hexahydrate (Zn(NO_3_)_2_·6H_2_O), hexamethylenetetramine, hydrochloric acid (HCl), sodium molybdate (Na_2_MoO_4_·2H_2_O), thioacetamide (CH_3_CSNH_2_), N,N-dimethylformamide(DMF), methylene blue, and anhydrous ethanol were purchased from Sinopharm Chemical Reagent Shanghai Co., Ltd.

### 2.2. Preparation of Samples

#### 2.2.1. Preparation of Polyimide Nanofibers

The nanofiber by electrospinning (high specific area and porosity) can be used as a template for growth of various nanomaterials [22]. In this experiment, polyimide nanofibers were prepared using a two-step method. First, 4.33 g PMDA and 3.97 g ODA were reacted in 35 mL of N, N-dimethylformamide (DMF) by polycondensation. The stirring of the solution was stopped after 8 h, and the precursor of polyamide acid (PAA) was obtained. The PAA nanofibers were prepared by electrospinning (KD Scientific 100, KD Scientific Inc., Holliston, MA, USA and Tianjin Dongwen 30 KVDC, Dong Wen High-Voltage Power Supply Corp., Tianjin, China) with the PAA solution (15%) at 15 kV with 15 cm from needle to collector. After electrospinning of the PAA solution, the electrospun PAA nanofibers were obtained. After thermal imidization, polyimide nanofibers were prepared.

#### 2.2.2. Growth of ZnO Seed Layer

First, the nanofibers were dipped and pulled coated multiple times to ensure that each nanofiber was loaded with a zinc acetate/ethanol solution. The fiber was immersed in the 0.005 mol/L Zn(Ac)_2_-CH_3_CH_2_OH solution for 60 min. After this, the soaking fiber was put on a petri dish to dry naturally until the ethanol evaporated. The fiber was then put into the ZnO seed growth solution to allow the surface of the fiber to infiltrate the seed solution. This process should be repeated several times in the experiment. Finally, the PI-ZnO composite fiber was decomposed by heating in an oven at 300 °C, and a uniform scale-like ZnO seed layer was obtained on the fiber.

#### 2.2.3. Growth of ZnO Nanorods

Next, the ZnO nanorod array is grown on the surface of PI fibers by the wet chemical method, and the growth environment needs to be calm and cannot be shaken. A 0.025 mol/L zinc nitrate aqueous solution and a 0.025 mol/L hexamethylenetetramine (HMTA) aqueous solution were mixed and stirred firstly. We put the mixed solution into a hydrothermal reactor. The PI fibers with the ZnO seed layer were soaked in a reactor for 4 h. At the same time, the reaction kettle is sealed and put in the oven at 90 °C for several hours. After taking out the sample, we washed it with deionized water many times and then dried it in an oven at 60 °C. Finally, the PI-ZnO composite fibers with a nanocomposite structure of fiber-nanorods were obtained. The surface of the PI-ZnO hybrid fiber became pale, which showed a pale-yellow color on the fiber surface.

#### 2.2.4. Growth of MoS_2_ Nanosheets

**Figure 1:**
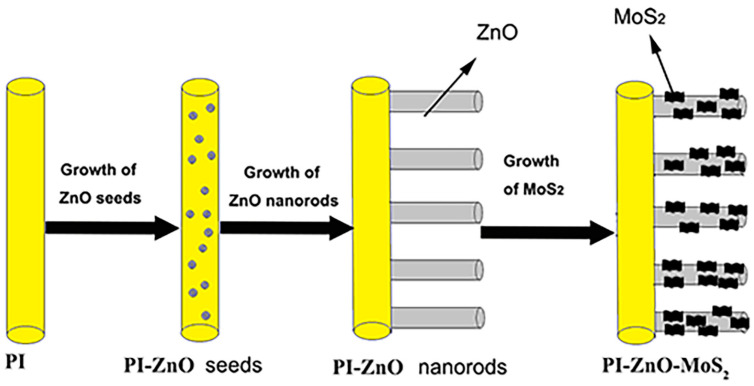
Scheme for the preparation of PI-ZnO-MoS_2_.

An amount of 30 mg of sodium molybdate and 60 mg of thioacetamide were dissolved in deionized water (20 mL). Then, they were added into a Teflon-lined stainless-steel autoclave. Then, 0.1 g PI fibers with ZnO nanorods were placed into the autoclave. Then, it was sealed and reacted at 200 °C for 24 h. After the reaction, the product was washed several times with deionized water and dried in a vacuum oven at 60 °C. Finally, the 0.14 g hybrid nanofibers with a composite structure of nanofibers–nanorods–nanosheets could be obtained. The fiber surface became darker and appeared matte black. The scheme for preparation of PI-ZnO-MoS_2_ composite nanofibers was shown in Figure 1.

### 2.3. Characterization

The morphologies of the as-obtained samples were observed using field-emission scanning electron microscopy (FE-SEM, Carl Zeiss Co., Ltd., Aachen, Germany (10 kV) and Hitachi S4800, Hitachi Corporation, Tokyo, Japan (15 kV)). X-ray photoelectron spectroscopy (XPS) measurements were carried out with an ESCALAB 250 photoelectron spectrometer (Thermo Fisher Scientific, Waltham, MA, USA) using Al Ka radiation. The crystal structure of the products was characterized by X-ray diffraction (XRD, Bruker D8 Advance diffractometer, Bruker, Bremen, Germany) using CuKa 1 radiation (λ = 0.15406 nm). UV–vis absorption spectra were obtained using a UV–vis spectrophotometer (UV-3600, Shimadzu, Tokyo, Japan). The UV–vis diffuse reflectance spectra (DRS) were obtained using UV–vis spectrophotometer (V-650, Jasco Corporation, Tokyo, Japan). The xenon lamp (PLS-SXE 300/300UV, Perfectlight Technology Co., Ltd., Beijing, China) with a wavelength of 380–780 nm was used to photocatalyze the reaction under visible light.

## 3. Results and Discussion

### 3.1. Morphology and Structure

**Figure 2:**
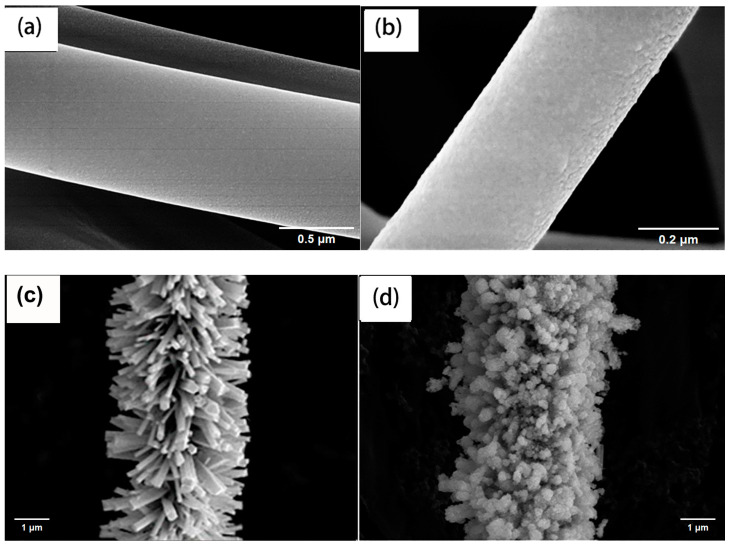
Field emission scanning electron micrographs of different composite fiber samples: PI nanofibers (**a**) and ZnO seed layer covering PI nanofibers (**b**), PI-ZnO nanocomposite nanofibers (**c**), PI-ZnO-MoS_2_ hybrid nanofibers (**d**).

The images in Figure 2 show pure PI nanofibers (Figure 2a) and a scale-like seed layer growing on the surface of the PI fibers (Figure 2b) with a size of around 35–50 nm. Figure 2c shows the structure of ZnO nanorod array after the hydrothermal reaction at 90 °C. The ZnO nanorod array obtained after 6 h of reaction shows an array distribution on the surface of the PI fiber. Due to the cylindrical fiber morphology, ZnO forms a circular isotropic distribution around the fiber surface, and the distribution direction is approximately perpendicular to the fiber surface. The diameter of ZnO on the surface of the PI fiber is about 35~150 nm, and the length is about 800–1000 nm. Morphologically speaking, ZnO nanorods are scattered on the fiber surface, occupying the fiber surface which is mainly determined by the uniformity of the growth of the ZnO seed layer. In Figure 2d, the MoS_2_ nanosheets are well supported on the ZnO nanorods. The nanosheets did not form a larger number of agglomerations on the nanorods, and the surface of this multi-stage structure composite fiber provided a vast space for the load of MoS_2_.

**Figure 3:**
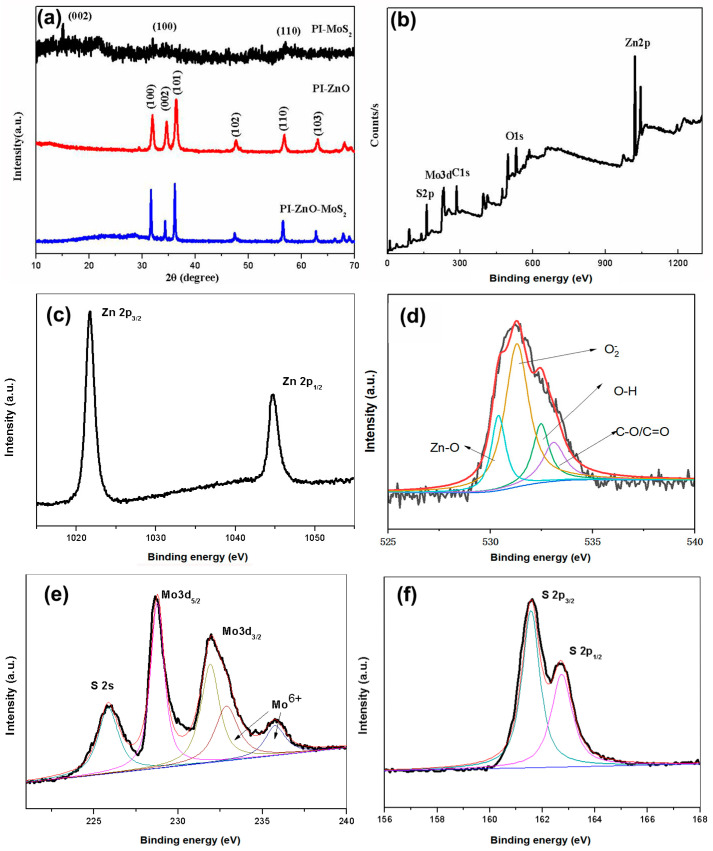
XRD patterns of different composite nanofibers (**a**). XPS spectra of the PI-ZnO-MoS_2_ nanocomposite structure of full range (**b**), Zn 2p (**c**), O 1s (**d**), Mo 3d (**e**), and S 2p (**f**).

As shown in Figure 3a, the PI-MoS_2_, PI-ZnO, and PI-ZnO-MoS_2_ nanocomposites were characterized using X-ray diffraction (XRD). The following strong XRD peaks at 2θ = 31.77°, 34.42°, 36.25°, 47.54°, 56.6°, and 62.86° can be clearly observed and are consistent with the (100), (002), (101), (102), (110), and (103) planes of ZnO. The phase of ZnO is indexed by the hexagonal wurtzite phase of ZnO (JCPDS Card No. 36-1451). For PI-MoS_2_ samples, the detected peaks can be assigned to (002), (100), and (110) planes in the hexagonal MoS_2_ (a = b = 0.316 nm, c = 1.230 nm, JCPDS card number 37-1492) [14]. These diffraction peaks are similar to the diffraction peaks of MoS_2_ nanosheets synthesized by another report [23]. The PI-MoS_2_ has very weak peaks, which is also less obvious in PI-ZnO-MoS_2_. The crystallinity of the PI-ZnO-MoS_2_ was enhanced after the composite formation due to this possible reason, which thermal annealing reduced the compressive stresses to stress-free state [24].

The X-ray photoelectron spectroscopy (XPS) characterizes the chemical composition and valence state. The full-range XPS spectrum of PI-ZnO-MoS_2_ (0~1250 eV) is shown in Figure 3b. Figure 3c shows Zn 2p 3/2 and Zn 2p 1/2 peaks at 1021.8 and 1044.8 eV, respectively, which are attributed to Zn^2+^ from ZnO. The difference of these energy levels (Δ = 23) from pure ZnO is attributed to strong interfacial interactions between ZnO and MoS_2_ [20,25]. In Figure 3d, the O 1s core energy level spectrum recorded on the sample is asymmetric with four peaks: 531.1 eV, which is ascribed to the adsorbed oxygen ion $O_{2}^{-}$; 530.1 eV, which corresponds to Zn-O; 532.0 eV, which shows the presence of O-H; and 533.2 eV, which can be attributed to C-O/C=O from PI nanofibers [26]. As shown in Figure 3e, the high-resolution XPS spectrum shows Mo 3d 3/2 and Mo 3d 5/2 peaks in the MoS_2_/ZnO heterojunction. The binding energies are located at 228.8 and 232.3 eV, indicating that the Mo element is currently in the chemical state of Mo^4+,^ and the compound showing the binding energy is MoS_2_. In addition, there is a peak at the binding energy of 226.0 eV that can be assigned to S 2s as well as a peak at the binding energy of 236.0 and 233.0 eV that corresponds to Mo^6+^ of MoO_3_ owing to the small portion of Mo oxidation [27]. Figure 3f shows that the binding energies of S 2p 3/2 and S 2p 1/2 are 161.6 and 162.8 eV, respectively. Based on these results, the nanostructures synthesized on the surface of PI nanofibers can be definitively identified as ZnO-MoS_2_ nanostructures.

**Figure 4:**
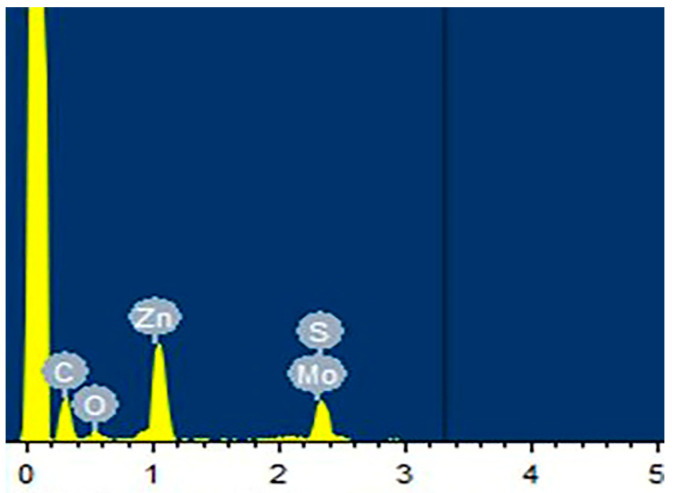
EDS spectra of PI-ZnO-MoS_2_ composite nanofibers.

From the EDS spectrum of the PI-ZnO-MoS_2_ multi-stage composite structure nanofiber, we can see that the peak of Zn is the highest, followed by S, Mo, and O with the lower peak in Figure 4. It can be seen that the selected points are rich in Zn elements and contain a small amount of O and C elements. This shows that ZnO is the main component in large grains, for the ZnO-rich region, while the other doping content is very small. In this way, we can know that rich ZnO nanorods were grown onto the fibers during synthesis. Near Mo and S elements, there are few other doped elements, that is, MoS_2_ nanosheets are grown on the nanorods, and this is the desired structure.

### 3.2. Photocatalytic Activity the As-Prepared Photocatalysts

Finally, we conducted an evaluation of the performance of PI-ZnO-MoS_2_ in photocatalytic degradation of methylene blue (MB) at room temperature. Its performance can be judged by the concentration of methylene blue after visible light irradiation at different times. The solution color of methylene blue has changed from blue to colorless with the increase of the time under visible light. Its performance corresponds to the decrease of concentration of methylene blue. The peak value occurs at about 663 nm, which corresponds to the specific absorption peak of MB.

The degradation-remainder can be reacted with the following Formula (1):

(1) $$D-R=C/C_{0}$$

C_0_—the initial methylene blue content;
C—the content of methylene blue corresponding to the increase of the photocatalytic time;
D − R—residual degradation rate.

**Figure 5:**
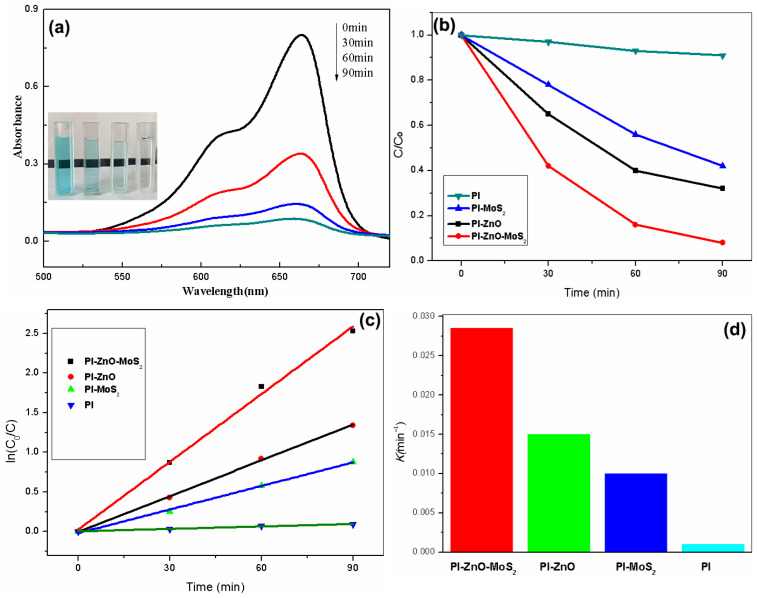
Visible light absorption spectra of methylene blue (MB) as a function of visible light irradiation time (λ = 380–780 nm) in the presence of the PI-ZnO-MoS_2_ composite nanofibers (**a**); time-dependent visible light absorbance of the MB solution after photodegradation by different membranes (**b**). The ln(C_0_/C) versus time (**c**) and reaction rate constants (*k*) of the MB photodegradation by different catalysts (**d**).

The photocatalytic performance of PI-ZnO-MoS_2_ was evaluated at room temperature as shown in Figure 5. Before exposure to visible light, the solution was kept in the dark for 30 min to establish the adsorption/desorption balance. In Figure 5a, it shows the UV–vis spectra of MB (4 mg/L) solution after irradiation in the presence of PI-ZnO-MoS_2_ (0.02 g). The characteristic absorption band of MB at 663 nm was measured every 30 min by an ultraviolet–visible spectrophotometer. With the increase of the irradiation time, the intensity of the characteristic absorption band of MB at 663 nm has significantly weakened. At the same time, the solution changed from blue to colorless after 90 min of exposure, thus indicating that MB molecules gradually decomposed under visible light irradiation. The degradation efficiency is C/C_0_, where C is the absorption of the main peak of MB at 663 nm at time t, C_0_ corresponds to the initial concentration (after reaching the adsorption/desorption equilibrium).

**Figure 6:**
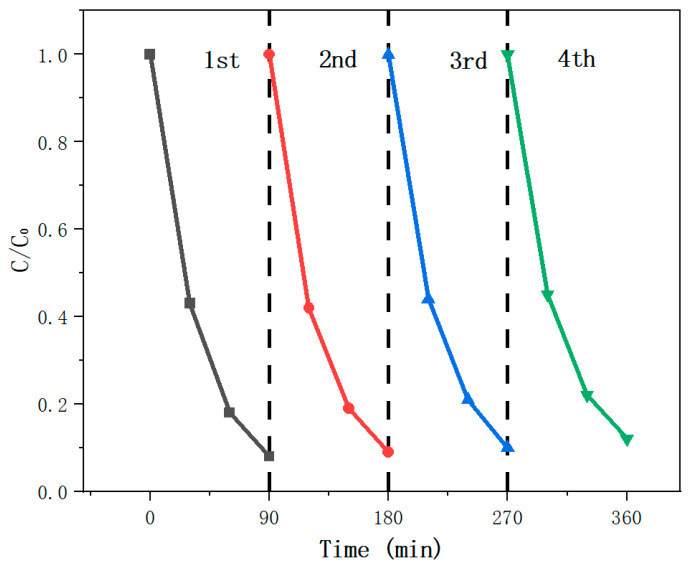
Stability and repeatability of PI-ZnO-MoS_2_ for MB photodegradation.

As shown in Figure 5b, we investigated the degradation rate of dye in various materials under visible light irradiation. The results show photocatalytic performance of PI, PI-MoS_2_ PI-ZnO, and PI-ZnO-MoS_2_ under visible light for the degradation of methylene blue. The pure PI nanofibers hardly showed any photocatalytic performance. However, the PI-ZnO and PI-MoS_2_ nanofibers could degrade the MB dye up to 68% and 59% after 90 min of irradiation, respectively. The PI-ZnO-MoS_2_ composite material can decompose about 92% of methylene blue in 90 min under visible light irradiation. Compared with the PI-ZnO composite fibers, the PI-ZnO-MoS_2_ can significantly improve the photodegradation efficiency. To evaluate the photocatalytic efficiencies of these catalysts, the corresponding apparent reaction rate constants (*k*) of the MB degradation kinetics were calculated by using the equation ln(C_0_/C) = *k*t. Figure 5c shows the plots of ln(C_0_/C) vs. t under four materials, where C_0_ and C denote the concentrations of MB at t = 0 and t = t, respectively. The ln(C_0_/C) value increased linearly with t, indicating that the photocatalytic reaction follows a pseudo-first-order rate law. For the pure PI, the MB degrades at a relatively low reaction rate with *k* = 0.001 min^−1^. For other catalysts (PI-MoS_2_, PI-ZnO and PI-ZnO-MoS_2_), the corresponding degradation rates with *k* = 0.01 min^−1^, 0.015 min^−1^, and 0.0285 min^−1^ were obtained, respectively, and the PI-ZnO-MoS_2_ exhibits prominent catalytic activity (Figure 5d). Based on it, we conclude that the PI-ZnO-MoS_2_ composite can promote light absorption efficiency, reduce e–h reorganization, and enhance photogenerated charge separation [28]. Furthermore, after four cycles, the degradation efficiency remains 88%, suggesting the good repeatability and stability of PI-ZnO-MoS_2_ as an excellent photocatalyst (Figure 6), which is easy to recycle compared with other nanomaterials (nanoparticles, carbon nanotubes, graphene, et al.) as skeletons. The mechanism of visible-light photocatalysis of the PI-ZnO-MoS_2_ will be explained in the following sections.

### 3.3. Mechanism of the Experiment

The hydrothermal preparation of ZnO nanorods are widely used with Zn (NO_3_)_2_ and HMTA. In this case, Zn (NO_3_)_2_ provides Zn^2+^ for synthesis of ZnO nanorods [29]. It is known that HMTA is a highly water soluble, non-ionic tetradentate cyclic tertiary amine, which provides OH ions for the crystallization of ZnO and forms ligand with Zn^2+^ in the solution [30]. HMTA can slowly decompose to provide a gradual and controlled supply of ammonia, which can form ammonium hydroxide as well as complex Zn^2+^ to form Zn(NH_3_)_4_^2+^. The slow release of hydroxide can significantly affect the kinetics of the reaction since dehydration of the zinc hydroxide intermediates controls the growth of ZnO. HMTA and ammonia can also coordinate with ZnO crystals to inhibit the growth of certain surfaces. The reaction process is shown in (2)–(7). Compared with the high PH value environment, if HMTA is decomposed quickly to produce a large number of OH^−^ in a short time, the Zn^2+^ in the solution will precipitate quickly. The reactants are then rapidly consumed, and the orientation of the ZnO nanorods is stopped. In the reaction (3)–(5), the decomposition products of NH_3_ and HTMA play a very important role. First, it is a basic environment required for the reaction, that is, the necessary Zn (OH)_2_. Secondly, Zn (OH)_2_ is dehydrated in an oven and broken to generate ZnO nanorods.

(2) $$HMTA+6H_{2}O↔4NH_{3}+6HCHO$$

(3) $$NH_{3}+H_{2}O↔NH_{4}^{+}+OH^{-}$$

(4) $$Zn^{2+}+4NH_{3}↔[Zn(NH_{3}{)}_{4}{]}^{2+}$$

(5) $$[Zn(NH_{3}{)}_{4}{]}^{2+}+2OH^{-}↔ZnO+4NH_{3}+H_{2}O$$

(6) $$Zn^{2+}+2OH^{-}↔Zn(OH{)}_{2}$$

(7) $$Zn{\left(OH\right)}_{2}↔ZnO+H_{2}O$$

**Figure 7:**
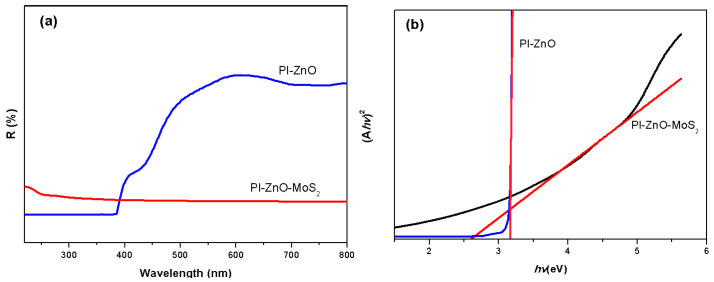
UV–vis DRS spectra of samples (**a**), and picture of (Ahν)^2^ versus hν for the samples (**b**).

In order to evaluate the effect of MoS_2_ nanoparticles on the optical properties of PI-ZnO-MoS_2_ nanocomposites, we used UV–visible diffuse reflectance spectrum (UV–vis DRS) analysis. Figure 7a shows the corresponding UV–vis DRS spectrum of the PI-ZnO and PI-ZnO-MoS_2_ samples. The absorption threshold of the sample is obtained by obtaining the UV–vis DRS curve from the tangent position of the extrapolated spectrum. In other words, ZnO nanoparticles have photocatalytic performance in the ultraviolet region due to their wide band gap (~3.3 eV). Similar to other wide bandgap semiconductors, ZnO is restricted to ultraviolet excitation, and there is little absorption in the visible range. Hence, it is desirable to develop a photocatalyst with an enhanced response from the UV to the visible spectral range. MoS_2_ exhibits strong absorption in the visible region. However, the fast recombination of photogenerated charge carriers and poor conductivity of MoS_2_ limits its application of photocatalytic process. As shown in Figure 7a, we can measure the threshold wavelength of the synthesized PI-ZnO-MoS_2_ sample is about 477 nm. Compared with pure ZnO, a red shift of the absorption edge to the visible region is observed in the nanocomposite. This is due to MoS_2_ being caused by strong light absorption in the visible light region, which indicates that there is an interface interaction between ZnO and MoS_2_.

Using the calculated band gap energy (Eg) equation of the sample:

(8) $${\left(Ah\nu \right)}^{2}=K(h\nu -Eg)$$

hν—photon energy (eV);
A—absorption coefficient;
K—constant;
Eg—band gap.

(Ahν)^2^ can be calculated by extrapolating the graph with the photon energy (Figure 7b). The PI-ZnO nanofibers have a band gap of ~3.1 eV. The band-gap energy decreased relative to other ZnO structures, which could be attributed to some crystal defects and broadening the light absorption based on their special brush-shaped structural features [31]. The band gap energy value of the PI-ZnO-MoS_2_ nanocomposite is calculated as ~2.6 eV, which is lower than the Boron–ZnO-MoS_2_ (3.18 eV) [32], ZnO/MoS_2_ (2.76 eV) [33], and ZnO/MoS_2_/rGO (2.86 eV) [34] composites from other reports.

A possible mechanism for the electromigration activity of composite materials is proposed based on experimental results. Compared with unmodified PI-ZnO nanofibers, the photocatalytic activity of the PI-ZnO-MoS_2_ has been obviously improved. This is mainly because the specific surface area of the PI-ZnO-MoS_2_ and the high separation efficiency of photogenerated electron–hole pairs are improved after growth of MoS_2_ nanosheets on the PI-ZnO. As can be seen from Figure 2d, the ultrathin MoS_2_ nanosheets were grown on the surface of PI-ZnO. Obviously, the specific surface area of the PI-ZnO-MoS_2_ is higher than that of PI-ZnO [35]. The MoS_2_ nanosheets can accept electrons and provide active sites for the degradation of MB, due to its quantum-confinement effect, which will benefit the sorption and desorption processes on the surface of the photocatalyst [36].

Furthermore, the PI-ZnO-MoS_2_ exhibited a stronger absorption (lower reflection) between 400 and 800 nm (Figure 7a). This result revealed that the integration of ultrathin MoS_2_ nanosheets with ZnO nanorods could significantly improve the light-harvesting ability of the composites. The PI-ZnO-MoS_2_ nanocomposites exhibited a red-shift absorption edge in comparison with the PI-ZnO, which indicates the addition of visible active MoS_2._ Based on the previous results, the corresponding band gap energy values were 3.1 and 2.6 for pristine PI-ZnO and the PI-ZnO-MoS_2_, respectively. Obviously, it is strongly evident that the composite material has a low band gap energy compared to the PI-ZnO. That is to say, the PI-ZnO-MoS_2_ nanocomposites have minimal band gap energy and a greater light absorption, which is responsible for improving the catalytic activity [37].

**Figure 8:**
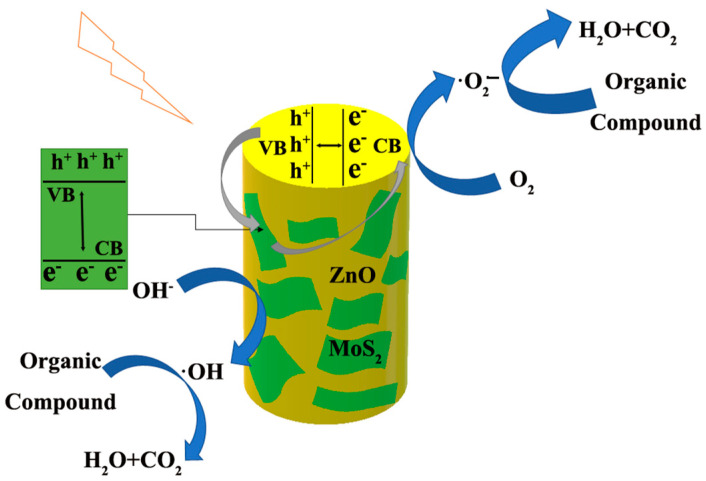
The proposed mechanism for photocatalytic degradation of MB solution in the presence of PI-ZnO-MoS_2_ nanofibers under visible light irradiation.

The mechanism is illustrated in Figure 8. The hybrid nanofibers potentially have a synergistic effect, where ZnO and MoS_2_ act together to separate electron-hole pairs and slow down the recombination rate. Due to the different work functions of the MoS_2_ (5.39 eV) and ZnO (4.7 eV) [38], after reaching the same Fermi level from the heterostructure, the conduction band of MoS_2_ nanosheets was situated above the conduction band of ZnO nanorods, and the valence band of ZnO nanorods was positioned below the valence band of MoS_2_ nanosheets [35]. Hence, the photogenerated electrons on the conduction band of the MoS_2_ can easily flow into that of ZnO. Similarly, the excited holes on the valence band of ZnO can easily flow into the valence band of MoS_2_. The separated electrons react with dissolved O_2_ to produce ·O_2_^−^. Finally, the e–h separation of light generation occurs on the surface of the composite material. Moreover, photogenic holes react directly with hydroxyl groups in water to produce hydroxyl radicals (·OH). The ·OH, and ·O_2_^−^ are used to degrade dyes [39]. The process can separate electrons and holes at interface, which increases the life of charge carriers and hinders recombination of the electron hole pairs [40,41]. As a result, high photocatalytic abilities are obtained.

## 4. Conclusions

PI-ZnO-MoS_2_ hybrid nanofibers were successfully fabricated by the growth of MoS_2_ nanosheets and ZnO nanorods on polyimide nanofibers using a simple hydrothermal method. Furthermore, SEM analysis proved that the MoS_2_ nanosheets were grown successfully on the brush-shaped PI–ZnO. The XRD analysis showed that the PI-ZnO-MoS_2_ has enhanced crystallinity. The XPS analysis results further confirm that MoS_2_ is successfully decorated on PI-ZnO. Compared with PI-ZnO (3.1 eV), the composite samples demonstrate low band gap energy (2.6 eV). The PI-ZnO-MoS_2_ nanocomposite fiber has good photocatalytic degradation efficiency, and its degradation efficiency of MB is 92% after 90 min. The heterojunction structure of ZnO-MoS_2_ enhanced the photogenerated charge separation. Compared with the ordinary surface modification of nanomaterials, the novel structures will have high specific surface area, high active sites, and low band gap energy for various functional applications, such as catalysis, gas separation, supercapacitors, sensors, etc.

## Data Availability

The data presented in this study are available on request from the corresponding author.
